# Predictors of death and production performance of layer chickens in opened and sealed pens in a tropical savannah environment

**DOI:** 10.1186/s12917-014-0214-7

**Published:** 2014-09-12

**Authors:** Aminu Shittu, Abdullahi Abdullahi Raji, Shuaibu A Madugu, Akinola Waheed Hassan, Folorunso Oludayo Fasina

**Affiliations:** Department of Theriogenology and Animal Production, Faculty of Veterinary Medicine, Usmanu Danfodiyo University, P.M.B 2254 Sokoto, Nigeria; Department of Pathology, Faculty of Veterinary Medicine, Usmanu Danfodiyo University, P.M.B 2254 Sokoto, Nigeria; Department of Production Animal Studies, University of Pretoria, Onderstepoort, 0110 South Africa; Agricultural Research Council of Nigeria, Plot 223D, Cadastral Zone B6, Mabushi, Abuja, Nigeria; Department of Animal Science, Faculty of Agriculture, Usmanu Danfodiyo University, P.M.B 2253 Sokoto, Nigeria

**Keywords:** Tropical climate, Egg production, Mortality, Survival modeling

## Abstract

**Background:**

Layer chickens are exposed to high risks of production losses and mortality with impact on farm profitability. The harsh tropical climate and severe disease outbreaks, poor biosecurity, sub-minimal vaccination and treatment protocols, poor management practices, poor chick quality, feed-associated causes, and unintended accidents oftentimes aggravate mortality and negatively affect egg production. The objectives of this study were to estimate the probability of survival and evaluate risk factors for death under different intensive housing conditions in a tropical climate, and to assess the production performance in the housing systems.

**Results:**

Daily mean mortality percentages and egg production figures were significantly lower and higher in the sealed pens and open houses (*P* < 0. 001) respectively. The total mean feed consumption/bird/day was similar for the open sided and sealed pens but the mean feed quantity per egg produce was significantly lower in the sealed pens ((*P* < 0.005). Seasons differently impacted on mortality with the hot-dry season producing significantly higher risk of mortality (61 times) and reduced egg production. Other parameters also differed except the egg production during the cold-dry season. Layers in sealed pens appear to have higher probability of survival and the Kaplan-Meir survival curves differed for each pen; ≥78 weeks old layer have higher probability of survival compared with the younger chickens and the 19–38 weeks age category are at highest risk of death (*P* < 0.001). The hazard-ratio for mortality of layers raised in sealed pens was 0.568 (56.8%).

**Conclusion:**

Reasons for spiked mortality in layer chickens may not always be associated with disease. Hot-dry climatic environment is associated with heat stress, waning immunity and inefficient feed usage and increase probability of death with reduced egg production; usage of environmentally controlled building in conditions where environmental temperature may rise significantly above 25°C will reduce this impact. Since younger birds (19–38 weeks) are at higher risk of death due to stress of coming into production, management changes and diseases, critical implementation of protocols that will reduce death at this precarious period becomes mandatory. Whether older chickens’ better protection from death is associated with many prophylactic and metaphylactic regimen of medications/vaccination will need further investigation.

## Background

Commercial egg-type poultry production is relatively low in northern Nigeria compared with the southern zones in view of a number of factors including but not limited to the climatic conditions which aggravate mortality and negatively affect egg production percentages. Other factors including severe disease outbreaks, poor biosecurity, sub-minimal vaccination and treatment protocols, poor management, practices, poor chick quality, feed-associated causes, and unintended errors and accidents similarly influence production [1]. Laying hens are female chickens which are raised primarily for the purpose of commercial egg production. These birds and the breeder flocks are particularly at a higher risk of production losses, stress and pecking, higher disease incidents, inclement weather conditions and death due to long term exposures (≥72 weeks) to these factors on farms compared to meat type chicken [2].

A negative association has been established between mortalities and net profits associated with lower egg productions [3,4], and a mortality of up to 4% during weeks 1–8, 15% during rearing (9–20 weeks) and 12% during the laying period (21- ≥72 weeks) has been established as standards for the industry in a tropical climate [5]. Ghodasara and colleagues [6] have broadly classified mortality in layer hens into three viz those associated with brooding stage (26.23%), growing stage (24.56%), and laying period (49.21%).

Diseases that have negatively impacted on production and increase mortality in layer hens include amongst others the following: Infectious bronchitis (up to 67% mortality and production losses) [7,8]; Newcastle disease (between 51.5 and 60% mortality and 15% production losses) [9-11]; Coccidiosis (between 35.26 and 51.38% mortality and production losses respectively) [4,12]; Infectious Bursal Disease (up to 40.4% mortality and production losses) [13,14].

Others include Fowl typhoid, fowl cholera, fowl pox, infectious laryngo-tracheitis, Marek’s disease, Mycoplasma infections, infectious coryza, egg prolapse, aflatoxicoses, necrotic enteritis and *E. coli* infections and these can contribute between 1 – 20% mortality and egg production losses of up to 50% based on severity [2,15-23].

Meanwhile, certain other highly fatal diseases with transboundary and trade limitation potentials like the highly pathogenic avian influenza particularly the H5 and H7 subtypes will cause between 80-100% mortality, with egg production down to near zero and a follow-up policy of depopulation.

The housing system has been confirmed to have major impact on mortality and production based on previous studies [24,25]. In Sweden, Fossum and colleagues [24] had confirmed that with a change from the conventional battery-cages to litter-based system, the submission of mortality from layer chicken farms increased, and Gerzilov et al., [25] in their works in Bulgaria found out that mortality also spiked in young flocks on the litter compared to other housing systems and tend to normalize as the birds grow older. In the current study, we evaluated the effect of open-sided and sealed intensive housing systems, ages of birds in lay and seasons on production performance and risk of death in layer chickens under a tropical environment.

## Results

### Descriptive statistics

The dataset comprised of 2,783 rows of observations, which represents the cumulative number of days from the initial mortality records (January 2010) to the end of study (December 2010) for all the 8 pens. Although the pen sizes remained the same, the stocking density and mean number of laying birds per pen differed. Specifically, the mean number of laying birds per pen were 8,414 (11.21%), 7,874 (10.49%), 7,452 (9.93%), 7,561 (10.07%), 6,447 (8.59%), 12,107 (16.13%), 14,149 (18.85%), and 11,072 (14.75%) for pen 1, 2, 3, 4, 5, 6, 7 and 8 respectively. Significant differences exist in total numbers of birds per pen initially housed and the final numbers taken out of each pen (Table 1). The sealed pens have significantly higher numbers of chickens placed compared to the open pens (*P* < 0.001).

**Table 1:** **Descriptive statistics of layer chickens’ mortality, egg production and feed consumptions**

**Pens**	**Chicken population per house (number)**			**Mortality in percentages**			**Egg production percentages**			**Total feed/layer/day (g)**			**Total feed/egg laid (g)**		
	**Mean ± SD**	**Min.**	**Max.**	**Mean ± SD**	**Min.**	**Max.**	**Mean ± SD**	**Min.**	**Max.**	**Mean ± SD**	**Min.**	**Max.**	**Mean ± SD**	**Min.**	**Max.**
House 1	8414 ± 992	6791	10354	0.17 ± 0.25	0	2.07	60 ± 11	25.00	82.13	116 ± 3	105	128	202 ± 50	137	483
House 2	7874 ± 650	4756	9753	0.20 ± 0.27	0	1.83	59 ± 12	25.20	83.90	117 ± 5.7	95	168	210 ± 63	136	593
House 3	7452 ± 1713	5927	11010	0.16 ± 0.23	0	2.03	63 ± 10	30.90	81.20	117 ± 3.5	114	155	195 ± 44	141	396
House 4	7561 ± 2013	5102	11199	0.16 ± 0.19	0	1.27	67 ± 11	31.90	85.50	116 ± 3.2	107	126	180 ± 39	135	376
House 5	6447 ± 1834	2666	9627	0.18 ± 0.28	0	3.03	57 ± 10	28.10	79.30	116 ± 4.1	106	125	215 ± 51	146	434
House 6	12107 ± 602	11090	13429	0.04 ± 0.04	0	0.22	66 ± 10	43.40	86.00	119 ± 14	104	217	183 ± 29	134	277
House 7	14149 ± 625	12670	15200	0.06 ± 0.05	0	0.34	68 ± 17	4.30	89.30	116 ± 8.8	86	166	211 ± 194	132	1970
House 8	11072 ± 1198	7707	12454	0.12 ± 0.13	0	1.03	74 ± 7.0	46.40	86.10	116 ± 3.1	75	121	160 ± 18	95	247
Open houses	**7573 ± 1661**	**2666**	**11199**	**0.18 ± 0.24**	**0**	**3.03**	**61 ± 12**	**25.00**	**85.50**	117 ± 4.0	95	168	**200 ± 51**	**135**	**593**
Sealed houses	**12614 ± 1367**	**9647**	**15200**	**0.07 ± 0.08**	**0**	**1.03**	**69 ± 13**	**4.30**	**89.30**	117 ± 10	75	217	**187 ± 123**	**95**	**1970**
										(NS)			(**)		
	(***)			(***)			(***)								

The total sample population was 83,033 layer chickens. All birds in each house were included for analysis. (***) Significant at *P* ≤ 0.001; (**) Significant at *P* ≤ 0.005; (NS) = Not significant.

Bold data in Table 1 indicated combined data for all open and closed houses.

Although, the mean mortality percentages were lower that the standards for the industry (12% from 20 to 72 weeks), the values obtained here exclude those birds removed due to ill health, poor performance and aggressive behaviour all of which are removed by culling. The mean mortality percentages were significantly lower in the sealed pens (0.07%) compared with the open houses (0.18%) (*P* < 0. 001). Contrastingly, the egg production figures were significantly higher in the sealed pens compared to the open-sided ones (*P* < 0. 001, Table 1). The total mean feed consumption per bird per day was similar for the open sided and sealed pens but the mean feed quantity used to produce an egg was significantly lower in the sealed pens ((*P* < 0.005, Table 1).

With respect to seasons, mortality was significantly higher in the hot-dry season compared with cold-dry and warm wet seasons with significantly reduced egg production, increased per day feed consumption and higher feed consumption per egg produced (Table 2). There were significant differences exist in the parameters between the open and sealed pens for all seasons except for the egg production during the cold-dry season (Table 2). Specifically, mortalities were significantly higher in the open pens during the cold-dry, hot-dry and warm-wet seasons. Egg production was also significantly lower in the open pens for all seasons except the cold dry season. There were marginal but significant increase in the total feed consumed per bird as well as total feed consumed per egg laid in the open houses during the hot-dry season but these trends were reversed during the cold-dry season. A specific trend can not be established for the warm-wet season based on this study.

**Table 2:** **Comparison of the effect of season on mortality, egg production and feed consumption**

	**Mortality in percentages ± SD**			**Egg production percentages ± SD**			**Total feed/layer/day ± SD (g)**			**Total feed/egg laid ± SD (g)**		
	**Open houses**	**Sealed houses**		**Open houses**	**Sealed houses**		**Open houses**	**Sealed houses**		**Open houses**	**Sealed houses**	
Cold dry season	0.14 ± 0.25	0.09 ± 0.11	**	65.27 ± 11.27	65.48 ± 18.96	NS	114.57 ± 2.86	117.10 ± 17.12	*	182.32 ± 41.32	226.0 ± 208.96	**
Hot dry season	0.35 ± 0.31	0.08 ± 0.11	**	50.16 ± 8.78	71.49 ± 7.01	**	120.14 ± 5.21	117.18 ± 3.52	**	248.84 ± 57.71	165.47 ± 16.71	**
Warm wet season	0.09 ± 0.08	0.06 ± 0.06	**	65.08 ± 8.54	70.30 ± 9.02	**	115.91 ± 1.76	116.39 ± 2.24	**	181.73 ± 28.37	168.63 ± 24.65	**
Cold dry season (O + S)	0.12 ± 0.21		**	65.35 ± 16.63		**	115.52 ± 10.79		**	198.80 ± 133.71		**
Hot dry season (O + S)	0.25 ± 0.29			58.16 ± 13.17			119.03 ± 4.86			217.57 ± 61.77		
Warm wet season (O + S)	0.08 ± 0.07			67.04 ± 9.08			116.09 ± 1.97			176.82 ± 27.76		

*Significant at *P* ≤ 0.05; **Significant at *P* ≤ 0.001; NS = Not significant.

(O + S) = Open and sealed houses combined for the analysis.

**Figure 1:**
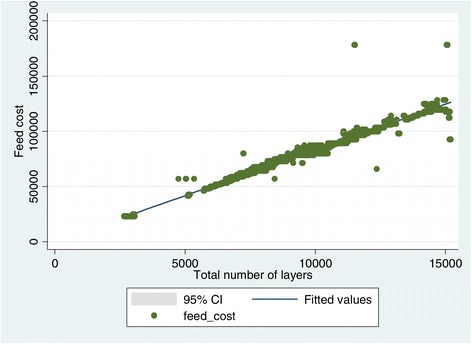
**Correlation curve between bird population and feeding costs.**

**Figure 2:**
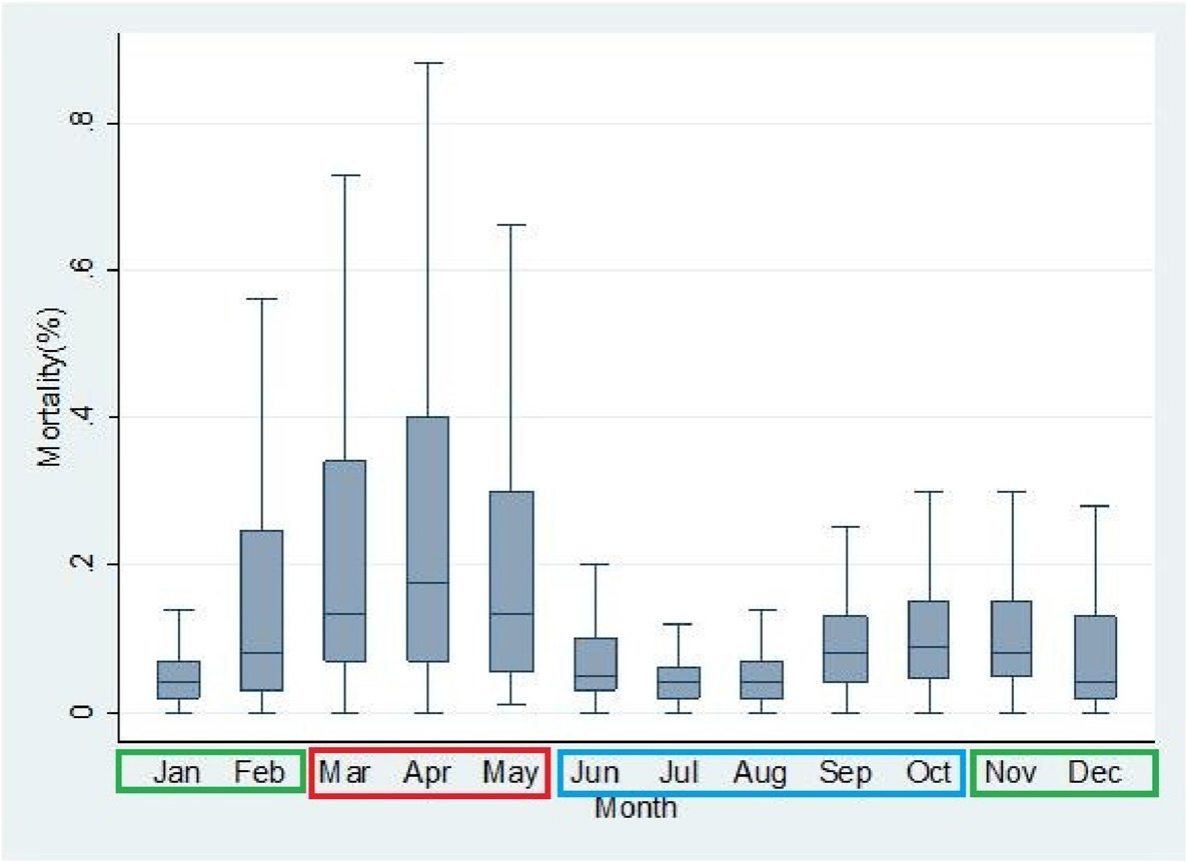
**Box plots of mortality percentages per month based on available data.**

**Figure 3:**
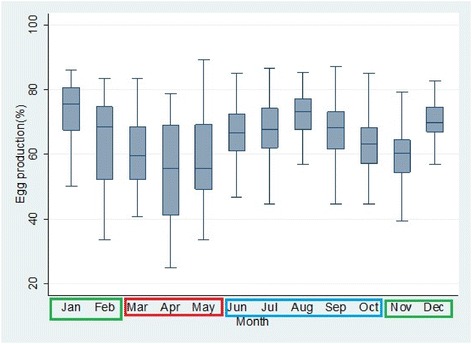
**Box plots of egg production percentages per month based on available data.**

Feed cost grew linearly with the rising populations of chickens stocked per house, however outlier effects were observed at certain data points due to uncertainties like sudden increase in feed prices and illness that forced reduced consumptions and consequent reduced costs per house (Figure 1). The months of February to May which corresponds with the hot-dry seasons had significantly spiked mortality (Figure 2) with reduced egg production (Figure 3).

### Survival and risk factors associated with layer poultry mortality

**Figure 4:**
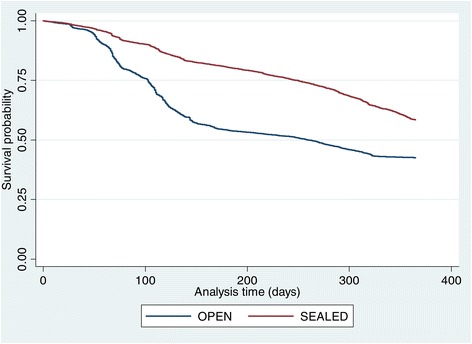
**Kaplan-Meir survival curves for open versus sealed pens.**

**Figure 5:**
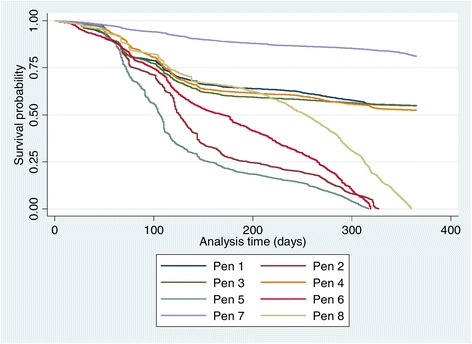
**Kaplan-Meir survival curves for individual open and sealed pens.**

**Figure 6:**
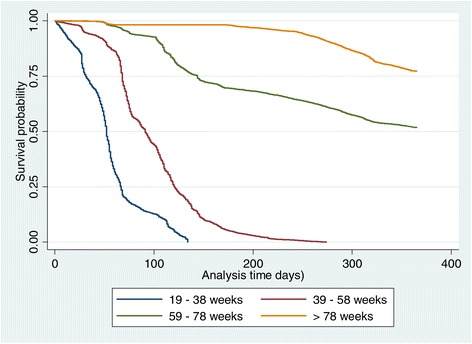
**Kaplan-Meir survival curves for all age categories of chickens included in the study.**

Using days as survival time and pen type (open and sealed), pen number (1–8) and age of layers (in weeks) as covariates, layers in sealed pens appear to have higher probability of survival (Figures 4 and 5) compared with those in the open pens. Differences exist among the Kaplan-Meir survival curves for each pen with pen number 2 & 5 (open), and 6 & 8 (close) having fewer probabilities of survivors towards the end of the curve. Pen 7 has the highest survival rates and is the pen with the highest stocking density in the farm. In addition, survival probabilities of layer poultry by their age revealed that layer birds ≥78 weeks old will have higher probability of survival compared with the younger chickens and the category at the highest risk of death are the layers in the age category 19–38 weeks (Figure 6).

All of the five predictors screened at the univariable level were taken forward for consideration in the multivariable Cox proportional hazard regression model. The small confidence intervals and low *P*-values obtained during the multivariable analysis support the huge size of dataset used in this study (Table 3). The final models revealed that all the five variables tested (season, pen type, age, stocking density and feed consumption) were associated with the probability of death among layers. The HR for mortality of layers raised in sealed pens was 0.568 (56.8%) compared with the open-sided penned layers. This ratio was assumed to be constant over the 12 months period.. The old layers ≥78 weeks are significantly less likely to die compared with the newly stocked young birds (19–38 weeks) (*P* < 0.001). Probability of mortality during the hot-dry season was 61 times compared with the cold-dry season which supports the descriptive analyses in Tables 1 and 2. The HR results for linear age, stocking density and feed consumption does not appear to increase the risk of mortality in layer birds.

**Table 3:** **Multivariable Cox-proportional hazard model, with pen as a shared frailty, to determine covariates of time to poultry layer mortality in the Savannah region of Nigeria**

**Factors**	**Level**	**Hazard ratio (95% CI)**	**Standard error**	**z**	**P value**
Pen type	Open	Ref	Ref	Ref	Ref
	Sealed	0.568 (0.554 - 0.582)	0.007	- 45.660	< 0.001
Season	Cold-dry	Ref	Ref	Ref	Ref
	Hot-dry	61.499 (58.836 - 64.283)	1.389	182.360	< 0.001
	Warm-wet	12.013 (11.566 - 12.478)	0.232	128.470	< 0.001
Age (weeks)	19 - 38	Ref	Ref	Ref	Ref
	39 - 58	0.329 (0.316 - 0.341)	0.006	−57.420	< 0.001
	59 - 78	0.032 (0.031 - 0.034)	0.000	−155.070	< 0.001
	> 78	0.011 (0.011 - 0.012)	0.000	−180.330	< 0.001
Age (linear)	Nil	0.911 (0.911 - 0.911)	0.000	−206.860	< 0.001
Stocking density (linear)	Nil	1.053 (1.043 - 1.064)	0.005	10.100	< 0.001
Feed consumption (linear)	Nil	1.012 (1.011 - 1.013)	0.000	32.160	< 0.001

## Discussions

The stocking density varied widely in the analysis based on the observation of same house types but different flock populations resident in the buildings. There was an underutilization of full capacity of the different houses on the farm. Though we cannot immediately assess whether the stocking density directly impact on the production efficiency of the chickens and total egg laid but we confirmed that it did not influence the risk of mortality. Guo et al. [26] had confirmed that whilewhile stocking density does not affect egg production percentages and weight, it will cause increased feed consumption and lead to poorer feed efficiency. Other workers have suggested that strain of birds and stocking density impact on egg production, egg weight, egg output and mortality, and recommended a standard of 733 cm^2^ per hen under the tropical environment [27]. From our observation, the stocking densities obtained for the different houses appeared low compared with the standards (450 cm^2^ or 69.8 inch^2^) recommended for the ISA Brown breed. The sealed house with environmental controlled buildings had significant higher production figures with lesser mortalities due to the fact that the chickens were kept in more clement conditions since weather conditions were automatically managed.

The hot-dry season presented with extremely higher mortality figures compared to the other months and the risk of death during this period was also very high. This season might impact negatively on production performance due to associated heat stress, waning immunity and feed qualities and quantities consumed during this period. Although the feed consumptions during this same period was slightly higher than for the other periods, the manager did indicated that more feed tends to be wasted in these months and more water were taken that in the colder and warmer months. The FAO has demonstrated that at a temperature above 28°C, egg production will significantly wane both in quantity and quality [4]. During the hot dry period in North-west Nigeria, environmental temperature may rise up to 47°C. Anjum has similarly confirmed the effect of high environmental temperature on egg production in Pakistan [26].

It should be noted that an environmental temperature between 25-40°C will cause the bird to pant and may lead to heat stroke and eventual death [28]. The climatic data for Gusau, Zamfara State was consistent with the above assertion and the findings in the study. A spike in mortality was associated with the hot-dry months of February to May, the months when average minimum/maximum temperatures and relative humidity values were as follows: (Jan: Temp = 7-40°C, Rel. humidity = 19%; Feb: Temp = 10-41°C, Rel. humidity = 16%; March: Temp = 12-43°C, Rel. humidity = 14%; April: Temp = 15-44°C, Rel. humidity = 21%; May: Temp = 20-47°C, Rel. humidity = 37%; June: Temp = 18-46°C, Rel. humidity = 50%) [29]. The effect of the above observation is an increasing production cost per egg produced and reduced profitability. It will thus be necessary to mitigate the excessive effect of heat stress on these birds during these months.

Since the sealed pen type with environmental controls appear to reduce the risk of death in layer birds by about half, it will become important to promote this housing type in sub-Saharan Africa where climatic conditions get extreme at certain period of the year.

It is noteworthy to state that younger birds (19–38 weeks) are at higher risk of death compared with older chickens (Table 3). They are at least three times more likely to die compared with those in age 39–58 weeks or 91 times more compared with those in age category ≥78 weeks. Previous workers have suggested that between 52 and ≈ 100% of the lead causes of death (collibacillosis, flock cloacal cannibalism, coccidiosis and lymphoid leucosis) of layer chicken in Sweden is associated with chickens at young age of between 20 and 39 weeks [24]. While we did not evaluate the causes of death, those diseases and conditions mentioned above and egg prolapse, chronic respiratory disease, infectious coryza, toxicities have been determined as the lead cause of death elsewhere [6,24]. It remains to be determined if the same lead causes of death are responsible for the mortalities seen in the flock in this study. However, it will be critical to implement management protocols that will reduce death through other means at this precarious period of the bird life when they are exposed to stress of coming into production, changed environment, changed feed, and other forms of stressful conditions. While it is clear that the older birds were better protected from death, it is probable that the many prophylactic and metaphylactic regimen of medications/vaccination they have undergone within the last months were responsible for this observation.

## Conclusion

Environmental controlled sealed buildings positively influenced egg production percentages and reduced mortality in a hot humid tropical climate. The chickens eat less quantities, waste more feeds and has reduced production efficiency during the hot dry environmental temperatures. Since younger chickens (19–38 weeks) are at higher risk of death compared with older chickens, due to stress of coming into production, management changes and diseases, critical implementation of management protocols that will reduce death through other means at this precarious period becomes mandatory. Effort should be intensified to distinctly identify disease-associated and production/management-associated deaths in layer chickens as reasons for spiked mortality in layer chickens may not always be associated with disease. This is important in order to reduce the burden of drug administration in production animals.

## Methods

### Description of studied farm

The farm is located in Bakura (12°09′N, 5°54′E), a low poultry density area of the Savannah region of northwest Nigeria, away from human residential areas. The distance of the farm from the nearest highway is 5 km and approximately 13 km away from the neighbouring town. Each pen contained adequate number of cages to accommodate the population of chickens per house. The farm has 12 pens, 6 of which are open-sided pens and the remaining 6 are sealed pens. These pens are in three rows: The first row has 4 open pens, the second row has 5 with 2 open pens and 3 sealed and the last row has 3 sealed pens. The temperature and relative humidity control in the open pens are influenced directly by the supervening climatic conditions while the sealed pens are environmentally controlled automatically by thermostat-controlled monitors. All the pens were equipped with automatic 3-tiers battery cages with facilities including feeders, nipple drinkers, litter conveyor belts beneath each tier and a feed silo. The birds (ISA Brown) were placed in the house before the onset of lay at 13–14 weeks and remained therein till end of lay (90 weeks) in open pens and (100 weeks) in sealed pens. This variation in end of lay age is taken as the farm’s standard. The chickens were dewormed at week 8 and 14 and were vaccinated against the following pathogens: Mareks disease, Newcastle disease, Infectious bursal disease, Fowl pox, Fowl typhoid and Egg drop syndrome. The lighting program, the farm strictly adheres to the ISA Brown management protocols. The birds had 16 hours (maximum) of photoperiod as at the time the egg production reached 50% till the end of lay. Body weight, day and night length were considered in the determination of photoperiod length. In 2010, a prospective cohort study was conducted between 1st January and 31st December using longitudinal data from apparently healthy layer chickens from this regional commercial poultry farm. This farm largely supplies eggs and culled layers in northwest Nigeria. As at the time of data collection, only 5 open and 3 sealed pens were fully functional, giving a total of 8 pens used for the study. All records of daily, weekly and monthly activities were obtained through a data retrieval system on each chicken in the pens. Follow-ups documents and pathology reports/mortality records on each bird were obtained where necessary to exclude ambiguous data and iatrogenic causes of death within the period of study (365 days). All records of daily mortalities were also evaluated by either of the 2 resident poultry veterinarians to confirm their authenticities.

### Predictor variables

A set of predictor variables believed to influence production parameters and mortality in poultry were obtained through the literature search [19,24,30-33]. A summary and detailed description of each variables included in the dataset is shown in Table 4. Only variables that are putative covariates or biologically plausible for layer poultry performance were selected and included in our statistical modelling. These include: season (cold-dry, hot-dry, and warm-wet), pen type (open and sealed), age (in days and weeks), stocking density of layers per pen, and daily amount of feed consumption. Age, stocking density, and feed consumption were treated as either linear or categorical terms or both. Survival was estimated as the time between daily mortality recordings, calculated by subtracting the recent date of death from the previous date (24 hours apart). The cause of death was considered to be the final event regardless of the underlying cause.

**Table 4:** **Summary of variables used in statistical analyses for identifying covariates for layer poultry performance in opened and sealed housing systems in the Savannah region of Nigeria**

**Variable**	**Description**
Month	The month during which total daily egg production was recorded (January – December)
Season	The season during which total daily egg production was recorded (cold-dry, hot-dry or warm-wet). This variable was generated from the Month variable.
Pen_number	Unique identifier for pen (1 – 8)
Pen_type	Unique identifier for the type of pen (opened or sealed)
Age	Age of layers during which total daily egg production was recorded (19 – 101 weeks)
Total_layers	Total number of layers or stocking density on daily record
Total_dead	Total number of dead layers on daily record
Mortality	Percentage dead of layers on daily record
Feed_layer	Quantity of feed (gram) required per layer per day
Feed_egg	Quantity of feed (gram) required per egg production per day
Feed_required	Total feed required (kilogram) per layer per day
Actual_feed	The actual feed (kilogram) fed to layers per day
Total_eggs	Total number of eggs produced per day
Total_crates	Total number of crates of eggs produced per day
Egg_production	Percentage daily egg production

### Data analysis and model building

In the study, the unit of study is the pen and mortality rate is based on the number of dead chicken per house per day divided by the number of birds housed per pen. All statistical analyses were performed using Stata® v. 10 (StataCorp, College Station, Texas 77845 USA). Continuous variables were analysed using descriptive statistics: minimum, maximum, mean and median values. Daily mortality counts were modeled using survival analysis. The Kaplan-Meier estimator was used to estimate the probability of survival and median survival time and the log-rank test was used to compare survival curves.

Univariable Cox’s proportional hazard modelling was performed to identify potential risk/hazard factors from the five variables initially considered. Those variables that demonstrated a statistically significant association(s) at *P* < 0.20 in the univariable model for death or were biologically plausible in layer mortality were considered for inclusion in the multivariable model. Multivariable analysis, adjusted for confounding factors, was used to determine the hazard ratio (HR) for death. Variables were retained in the multivariable model if likelihood-ratio-test *P* < 0.05 [34]. The backward stepwise analysis was used in the Cox proportional hazard regression model, with shared frailty, which was fitted to assess the relationship of multiple predictors with the mortality of layers. The model as suggested by Therneau and Grambsch [35] is shown as:

$$ {\lambda}_i(t)={\lambda}_0(t)\cdot {e}^{x_i\cdot \beta +{Z}_i\cdot \omega } $$

While the random effects was in *Z*_*i*_ ⋅ *ω*.

It was expected that observations with equal value of a variable in the data that identifies the group are assumed to have shared frailty. Across groups, the frailties are assumed to be gamma-distributed latent random effects that affect the hazard multiplicatively, or, equivalently, the logarithm of the frailty enters the linear predictor as a random offset. The results for the Cox proportional hazard model were reported as HR with its corresponding *P*-values and 95% CI. For each covariate, HR relative to the chosen reference category was calculated in order to show their relationships.

### Animal ethics

Although the observational data does not involve the handling and manipulation of animals, we ensured that all the “Five Freedom of Webster” (Freedom from hunger or thirst, Freedom from discomfort, Freedom from pain, injury or disease, Freedom to express (most) normal behaviour, Freedom from fear and distress) were taken care of in the farm. Similarly, the project was approved by the Research Committee of the Faculty of Veterinary Medicine, Usmanu Danfodiyo University, Sokoto. We also obtained and got clearance from the management of the farm to proceed and publish the outcome of the research.
